# Waist-to-height ratio is an effective indicator for comprehensive cardiovascular health

**DOI:** 10.1038/srep43046

**Published:** 2017-02-21

**Authors:** Shiwei Shen, Yun Lu, Huajin Qi, Feng Li, Zhenhai Shen, Liuxin Wu, Chengjian Yang, Ling Wang, Kedong Shui, Weifeng Yao, Dongchang Qiang, Jingting Yun, Lin Zhou

**Affiliations:** 1 Wuxi No.2 People’s Hospital Affiliated to Nanjing Medical University, Wuxi, Jiangsu 214002, China; 2 The Taihu Rehabilitation Hospital of Jiangsu Province, Wuxi, Jiangsu 214086, China; 3 Jiangsu Provincial Research Center for Health Assessment and Intervention, Wuxi, Jiangsu 214086, China; 4 Zhongguancun Xinzhiyuan Health Management Institute, Beijing, 100142, China

## Abstract

The aim of this study was to determine the associations between cardiovascular health and the waist circumference (WC) and waist-to-height ratio (WHtR). A cross-sectional study was performed recruiting 26701 middle-aged Chinese men. Of the seven ideal cardiovascular health metrics, body mass index (BMI), total cholesterol (TC), blood pressure (BP), and fasting blood glucose (FBG) were found to increase with an elevation of the mean WC and WHtR. The mean WC and WHtR were significantly lower in the subjects with intermediate or ideal cardiovascular health than those with poor or intermediate health. After adjustment for age, the mean WC and WHtR decreased by 1.486 cm and 0.009 per 1-point increase in the cardiovascular health score, and 2.242 cm and 0.013 per 1-point increase in the number of ideal cardiovascular health metrics, respectively. The cardiovascular health score was negatively correlated with the WC (*r* = −0.387) and WHtR (*r* = −0.400), while the number of ideal cardiovascular health metrics was negatively associated with the WC (*r* = −0.384) and WHtR (*r* = −0.395). The cardiovascular health is correlated negatively with the WC and WHtR, and a stronger correlation existed between the cardiovascular health and WHtR than WC.

Cardiovascular disease has become a global public health concern^1^. The 2013 Report on Cardiovascular Diseases in China estimates that approximately 290 million people have cardiovascular diseases in China^2^, and obesity has become a major risk factor leading to the increase in the prevalence of cardiovascular diseases^3^. Notably, abdominal obesity, which is caused by the accumulation of visceral fat, has been identified as an independent risk factor for obesity-related diseases and death^4^. The waist circumference (WC) and waist-to-height ratio (WHtR) are not only effective indicators of abdominal obesity, but also more effective parameters predicting risk factors for cardiovascular diseases^5^,^6^. Ideal cardiovascular health, which was proposed by the American Heart Association (AHA) in 2010, has been shown to be protective against cardiovascular and cerebrovascular diseases^7^,^8^,^9^,^10^,^11^,^12^. In the current study, we determined the associations between cardiovascular health and the WC and WHtR among middle-aged men in southeastern China to provide evidence for the development of preventive and control strategies for cardiovascular diseases.

## Results

### Baseline cardiovascular health metrics

A total of 26701 subjects were enrolled in this study, and the subjects at 40–49, 50–59, and 60–64 years of age consisted of 45.4%, 41.2%, and 13.4% of the total study subjects, respectively. The percentages of the seven ideal health metrics were as follows: total cholesterol (TC), 69.0%; fasting blood glucose (FBG), 67.4%; body mass index (BMI), 50.6%; physical activity (PA), 45.9%; smoking status, 40.5%; blood pressure (BP), 22.8%; and salt intake, 15.7%. Of the seven cardiovascular health metrics, BMI, TC, BP, and FBG were shown to increase with elevation of the mean WC and WHtR (all *P* values < 0.05) (Table 1).

### Number of cardiovascular health metrics and the WC and WHtR

There were only 132 subjects (0.5%) with seven ideal health metrics, 595 subjects (2.2%) with 0 ideal health metrics, and 7383 (27.7%), 6126 (22.9%), and 5702 (21.4%) subjects with 3, 4, and 2 ideal health metrics, respectively. The WC and WHtR were shown to have a clear-cut decreasing trend with the increase in the number of ideal cardiovascular health metrics (Table 2).

### Ideal cardiovascular health score and the WC and WHtR

The ideal cardiovascular health score predominantly ranged between 7 and 11, and there were 3432 (12.9%), 4470 (16.7%), 4847 (18.2%), 4422 (16.6%), and 2924 (11.0%) subjects with ideal cardiovascular health scores of 7, 8, 9, 10 and 11, respectively. Overall, the WC and WHtR had a remarkable decreasing trend with the increase in ideal cardiovascular health score (Table 3).

### Cardiovascular health status and the WC and WHtR

There were 798 (3.0%), 15964 (59.8%), and 9939 (37.2%) subjects with inadequate, average, and optimum cardiovascular health, respectively. The WC and WHtR were significantly lower in the subjects with average cardiovascular health than subjects with inadequate cardiovascular health, while a lower WC and WHtR were found in the subjects with optimum cardiovascular health relative to subjects with average cardiovascular health (Table 4).

### Association of cardiovascular health with WC and WHtR

Correlation analyses showed that cardiovascular health score was negatively correlated with the WC (*r* = −0.387) and WHtR (*r* = −0.400), and the number of ideal cardiovascular health metrics was negatively associated with the WC (*r* = −0.384) and WHtR (*r* = −0.395), while cardiovascular health was also negatively correlated with the WC (*r* = −0.319) and WHtR (*r* = −0.330). Stronger associations between the cardiovascular health score, number of ideal cardiovascular health metrics, and cardiovascular health were detected with the WHtR than the WC (Table 5). 10 as the cut-off point of cardiovascular health score, i.e. cardiovascular health score greater than or equal to 10 was defined as ideal cardiovascular health and cardiovascular health score less than 10 was defined as non-ideal cardiovascular health. The result of ROC analysis showed that the area under the curve (AUC) of WC was 0.678 and AUC of WHtR was 0.684.

## Discussion

Since ideal cardiovascular health was first proposed and defined by the AHA in 2010, the prevalence of ideal cardiovascular health has been reported worldwide; however, the cardiovascular health metrics and scores vary as a function of country, race, region, economy, and lifestyle^8^,^9^,^13^,^14^,^15^,^16^.

In the current study, we found that 132 of 26701 middle-aged Chinese men (0.5%) exhibited ideal levels of all seven cardiovascular health metrics, and 595 subjects (2.2%) had 0 ideal health metrics. The results of this study validate a low prevalence of ideal cardiovascular health in Chinese adults. The TC (69.0%) and FBG (67.4%) had the highest proportion of ideal levels, while salt intake (15.7%) and BP (22.8%) showed the lowest percentage of ideal levels, which was similar to the previous studies reporting a daily salt intake of >12 g per person in most areas of China^17^,^18^. High-salt diet is considered one of the major risk factors for developing hypertension in China, therefore BP control and salt intake reduction are one of the top priorities for the prevention and control of cardiovascular diseases.

Our findings showed that among the seven cardiovascular health metrics, BMI, TC, BP, and FBG correlated positively with WC and WHtR (all *P* values < 0.05). In addition, the WC and WHtR had a remarkable decreasing trend with an increase in the number of ideal cardiovascular health metrics (both *P* values < 0.05), and the WC and WHtR were significantly lower in the subjects with intermediate or ideal cardiovascular health than subjects with poor or intermediate health (both *P* values < 0.05), demonstrating close associations between ideal cardiovascular health, number of ideal cardiovascular health metrics, and cardiovascular health score with the WC and WHtR.

In the current study, both the WC and WHtR exhibited a remarkable decreasing trend with the increase in ideal cardiovascular health score. After adjustment for age, a 1-point increase in the cardiovascular health score was associated with a 1.486 cm reduction in the mean WC and a 0.009 reduction in the mean WHtR, and a 1-point increase in the number of ideal cardiovascular health metrics was associated with a 2.242 cm reduction in the mean WC and a 0.013 reduction in the mean WHtR. Ambar Kulshreshtha, *et al*., found that individuals with intermediate or ideal cardiovascular health had a significantly lower risk of stroke than those with poor health^7^. In addition, a 1-point higher cardiovascular health score was associated with an 8% lower risk of stroke (hazard ratio, 0.92; 95% CI, 0.88–0.95)^7^. It is therefore suggested that the following control strategy should be implemented to reduce the prevalence of cardiovascular diseases: (1) The four cardiovascular health behaviors (smoking, body mass index, physical activity and salt intake) and three health factors (total cholesterol, blood pressure and fasting plasma glucose) should be improved to increase the cardiovascular health score and/or the number of ideal cardiovascular health metrics. (2) WC and/or WHtR should be maintained within the normal range for abdominal obesity control. Although the seven cardiovascular health metrics include BMI, but the WC and/or WHtR are effective parameters in measuring the accumulation of abdominal fat.

Excessive body fat accumulation may lead to an increase in the risk factors for cardiovascular diseases, such as hyperinsulinemia, insulin resistance, hypertension, and blood lipid abnormalities, thereby resulting in the development of cardiovascular diseases^19^,^20^. The WC and WHtR are effective parameters for measuring abdominal obesity and predicting the risk factors for cardiovascular diseases^5^,^6^; however, the predictive value of the WC versus WHtR remains controversial. It has been widely reported that the WHtR is superior to the WC and BMI in predicting the risk for cardiovascular diseases^21^,^22^,^23^,^24^,^25^,^26^,^27^,^28^,^29^. A follow-up study conducted by Gelber which recruiting 16000 men and 32000 women showed the strongest correlation between the WHtR, one of the parameters measuring obesity, and cardiovascular diseases^21^. And the results from another 11-year prospective study involving 45,000 women <60 years of age revealed that the WHtR was superior to the WC, and the WC was superior to waist-to-hip-ratio (WHpR) in predicting the risk of stroke^22^. Lucy and colleagues proposed that the WHtR is a more ideal tool (a 0.5 cut-off value) to predict cardiovascular diseases and diabetes^30^, while Ashwel *et al*. reported that the WHtR is superior to the WC and BMI in predicting the risk for cardiovascular diseases^29^. Mannucci, *et al*., consider that the WHtR was shown to be superior to the WC and WHpR for predicting hypertension and hyperlipidemia in a United States population^31^. Most China researches revealed that the WHtR is better than the WC and BMI in predicting blood lipid abnormalities in a Chinese population^32^,^33^,^34^,^35^. In addition, a recent study conducted in Korea showed that the WHtR is better than the WC, while the WC is better than the BMI in predicting the risk for coronary heart disease, thus suggesting that the WHtR is an indicator measuring abdominal obesity in clinical practice^36^. It has been widely reported that the WHtR has a satisfactory predictive value, which may be explained by the following reasons. The WC cannot be used to quantify or differentiate visceral fat and subcutaneous fat, and the WC may be affected by many factors, such as gender, height, age, race, region, economy, environment, and lifestyle, while the BMI can only be used to measure total body fat and cannot represent fat distribution, the use of BMI alone may overestimate the risk for developing cardiovascular diseases in the population with a high weight and many muscular tissues^37^. The WHtR, which comprehensively considers the impact of height and WC, varies little as a function of race, age, and gender, and is relatively stable^38^. Our findings showed stronger associations between the cardiovascular health score, number of ideal cardiovascular health metrics, and cardiovascular health status with the WHtR than the WC. It is therefore suggested that the WC should be replaced by the WHtR as a simple tool to measure abdominal obesity and predict cardiovascular risk factors in primary health care.

The WC and WHtR cut-offs for measuring adult abdominal obesity has been controversial until now. The AHA recommends a 102 cm WC for men and 88 cm for women^39^, and the World Health Organization (WHO) and International Diabetes Federation (IDF) recommend a 90 cm WC for men and 80 cm for women in Asian-Pacific populations^40^, while the Working Group on Obesity in China recommends an 85 cm WC for men and 80 cm for women^41^. A study by the Japan Society for the Study of Obesity defined an 85 cm WC for men and 90 cm for women, which was similar to the visceral fat mass, and a Korean study reported an 83.2 cm WC for men and 79.7 cm for women^42^. He and colleagues recommended a 0.5 WHtR in both mainland Chinese men and women^32^, while a 0.45–0.48 WHtR cut-off was recommended for Taiwanese populations^33^,^34^ and a 0.48 cut-off in both men and women living in Hong Kong^37^. Lucy *et al*. reported a 0.5 WHtR cut-off in both men and women, and proposed a health initiative that WC does not exceed one-half of the height^30^. In addition, a recent Korean study defined a 0.5 WHtR in men and 0.52 in women^36^. Our findings showed that a 90 cm WC and 0.5255 WHtR at a 7 cardiovascular health score, and a 84.79 cm WC and 0.4944 WHtR at a 10 cardiovascular health score, which is similar to previous studies^30^,^32^,^36^. We consider that different regions should develop a reasonable WC and WHtR cut-off point based on the local epidemiological study and an 85 cm WC cut-off and a 0.5 WHtR cut-off may reasonable to Jiangsu resident.

In summary, the results of this study demonstrate that the cardiovascular health score correlates negatively with the WC and WHtR, and a stronger association between the cardiovascular health score was detected with the WHtR than the WC. In addition, the WHtR is of great value in screening populations at high risk for abdominal obesity and cardiovascular diseases and predicting the risk for cardiovascular diseases.

## Methods

### Subjects

A cross-sectional study was performed. The men between 40 and 64 years of age receiving health examinations in our hospital from 1 January 2014 through 30 June 2015 were recruited, and all recruited subjects resided in the Suzhou, Wuxi, and Changzhou regions of southeastern China. The study exclusion criteria included the following: use of lipid-regulating drugs; a history of myocardial infarction or stroke; severe hepatic or renal insufficiency; or incomplete medical records. A total of 26701 patients met the appropriate criteria.

The study protocol was approved by the Ethics Review Committee of the Taihu Rehabilitation Hospital of Jiangsu Province, and the study was performed in accordance with the principles of the Declaration of Helsinki. Written informed consent was obtained from all participants following a detailed description of the purpose of this study.

### Questionnaire survey

Demographic and clinical characteristics were captured using a self-designed questionnaire, including age, residency, profession, smoking status, alcohol consumption, salt consumption, living habits, physical activity status, medical history of chronic diseases (hypertension, diabetes, coronary heart disease, stroke, and other cardiovascular diseases), and medications. The questionnaire was administered by well-trained medical professionals.

### Measurement of cardiovascular risk factors

All subjects had measurements of height, weight, waist circumstance (WC), systolic blood pressure (SBP), diastolic blood pressure (DBP), and body mass index (BMI). In addition, all participants fasted for 8–12 h, and 5 mL of venous blood was collected from the cubital vein the following morning. The serum levels of TG, total cholesterol (TC), HDL-C, and LDL-C were determined using the glycerol phosphate oxidase method, the oxidase method, an antibody-based homogeneous assay, and the homogeneous assay on a fully automatically biochemical analyzer (Hitachi 7600; Hitachi, Ltd., Tokyo, Japan), respectively.

### Definition of cardiovascular health

Based on the definition of cardiovascular health proposed by the AHA in 2010^13^, vegetable intakes were changed to salt intake in this study. Physical activity was defined as moderate-intensity aerobic exercise, including fast walking, running, bicycle riding, rope skipping, and swimming and the classification criterion of physical activity was adjusted.

In accordance with AHA definitions, 7 CVH metrics were classified into ideal, intermediate, and poor: (1) smoking: ideal (never or quit >1 year), intermediate (quit <1 year), and poor (current); (2) body mass index (BMI): ideal (<25 kg/m^2^), intermediate (25 to <30 kg/m^2^), and poor (≥30 kg/m^2^); (3) physical activity: ideal (physical activity ≥3 times a week, with >30 min each time or physical activity >90 min per week), intermediate (physical activity of <3 times a week, with <30 min each time or ≤ 89 min of physical activity per week), and poor (no extra physical activity except daily life and work activities); (4) salt intake: ideal(<6 g/d), intermediate (6–12 g/d), and poor (>12 g/d) based on responses to questions related to salt preferences; (5) total cholesterol (TC): ideal (untreated and <5.2 mmol/L [200 mg/dL]), intermediate (treated to <5.2 mmol/L or 5.2–6.2 mmol/L), and poor (>6.2 mmol/L [240 mg/dL]); (6) blood pressure (BP): ideal (untreated and <120/<80 mm Hg), intermediate (treated to <120/<80 mm Hg or 120–139/80–89 mm Hg), and poor (≥140/90 mm Hg); and (7) fasting plasma glucose (FPG): ideal (untreated and <5.6 mmol/L [100 mg/dL]), intermediate (treated to <5.6 mmol/L or 5.6–7.0 mmol/L), and poor (≥7.0 mmol/L [125 mg/dL]).

For each subject, the seven cardiovascular health metrics were scored as follows: 0, poor; 1, general; and 2, ideal. The sum of the scores of the seven cardiovascular health metrics was defined as the total cardiovascular health score, and cardiovascular health status was classified according to the total score, as follows: 0–4, inadequate; 5–9, average; and 10–14, optimum^14^.

### Statistics

The WC and WHtR were described as the mean ± standard deviation (SD), while the distribution of ideal cardiovascular health components and number of ideal cardiovascular health metrics were expressed as a number (proportion).

The associations between WC, WHtR and the cardiovascular health score were calculated using Pearson correlation analysis. The associations between WC, WHtR and the number of ideal cardiovascular health metrics, cardiovascular health status were calculated using Spearman correlation analysis. The receiver operating characteristic curve (ROC) was used to compare the predictive value of WC and WHtR in ideal cardiovascular health. All statistical analyses were conducted using SPSS version 16.0 (SPSS, Inc., Chicago, IL, USA), with a two-tailed *P*-value < 0.05 considered statistically significant.

## Additional Information

**How to cite this article**: Shen, S. *et al*. Waist-to-height ratio is an effective indicator for comprehensive cardiovascular health. *Sci. Rep.* **7**, 43046; doi: 10.1038/srep43046 (2017).

**Publisher's note:** Springer Nature remains neutral with regard to jurisdictional claims in published maps and institutional affiliations.

## Figures and Tables

**Table 1:** Ideal cardiovascular health metrics and WC, WhtR.

Metrics		n	%	WC		WHtR	
				Mean	S.D.	Mean	S.D.
Age^abcdef^	40–49	12124	45.4	86.75	8.197	0.5034	0.04737
	50–59	11008	41.2	87.59	8.250	0.5123	0.04756
	60–64	3569	13.4	87.26	8.496	0.5148	0.04985
Smoking Status^abcdef^	Ideal	10801	40.5	86.97	8.020	0.5078	0.04645
	Intermediate	829	3.1	85.51	8.399	0.4985	0.04799
	Poor	15071	56.4	87.39	8.422	0.5098	0.04906
Body Mass Index^abcdef^	Ideal	13519	50.6	82.12	6.472	0.4786	0.03697
	Intermediate	12038	45.1	91.45	5.902	0.5341	0.03356
	Poor	1144	4.3	101.62	5.599	0.5941	0.03236
Physical Activity^bcdef^	Ideal	12268	45.9	87.12	8.066	0.5086	0.04684
	Intermediate	11914	44.6	86.98	8.366	0.5070	0.04859
	Poor	2519	9.4	88.27	8.689	0.5161	0.05031
Salt Intake^abcdef^	Ideal	4201	15.7	87.93	7.555	0.5135	0.04366
	Intermediate	17660	66.1	86.23	8.364	0.5030	0.04850
	Poor	4840	18.1	89.90	7.824	0.5248	0.04569
Total Cholesterol^acdf^	Ideal	18425	69.0	86.64	8.306	0.5053	0.04825
	Intermediate	6856	25.7	88.31	8.026	0.5157	0.04650
	Poor	1420	5.3	88.36	8.260	0.5166	0.04776
Blood Pressure^abcdef^	Ideal	6082	22.8	83.61	7.969	0.4871	0.04629
	Intermediate	11883	44.5	87.17	7.893	0.5082	0.04583
	Poor	8736	32.7	89.63	8.069	0.5241	0.04626
Fasting Blood Glucose^abcdef^	Ideal	17990	67.4	86.30	8.188	0.5032	0.04748
	Intermediate	5108	19.1	88.30	7.873	0.5158	0.04546
	Poor	3603	13.5	89.85	8.445	0.5255	0.04905

Note: a indicate that the difference of WC between Ideal and Intermediate (40–49 y and 50–59 y) is statistically significant; b, the difference of WC between Intermediate and Poor (50–59 y and 60–64 y) is statistically significant; c, the difference of WC between Ideal and Poor (40–49 y and 50–59 y) is statistically significant; d, the difference of WhtR between Ideal and Intermediate (40–49 y and 50–59 y) is statistically significant; e, the difference of WhtR between Intermediate and Poor (50–59 y and 60–64 y) is statistically significant; f, the difference of WhtR between Ideal and Poor (40–49 y and 50–59 y) is statistically significant.

**Table 2:** The number of ideal cardiovascular health metrics and WC, WHtR.

The number of ideal metrics	n	%	WC		WHtR	
			Mean	S.D.	Mean	S.D.
0	595	2.2	94.04	6.978	0.5499	0.04031
1	2621	9.8	92.13	7.388	0.5377	0.04232
2	5702	21.4	90.08	7.602	0.5262	0.04419
3	7383	27.7	87.26	7.970	0.5092	0.04618
4	6126	22.9	84.56	7.859	0.4932	0.04536
5	3168	11.9	83.09	7.238	0.4841	0.04153
6	974	3.6	82.16	6.622	0.4784	0.03827
7	132	0.5	81.56	5.780	0.4773	0.03463

**Table 3:** The ideal cardiovascular health score and WC, WHtR.

Score	n	%	WC		WHtR	
			Mean	S.D.	Mean	S.D.
0	2	0.0	100.00	5.657	0.5837	0.01496
1	6	0.0	101.67	6.683	0.5830	0.03687
2	62	0.2	99.95	6.624	0.5857	0.04448
3	174	0.7	96.53	7.139	0.5666	0.04008
4	554	2.1	94.81	7.842	0.5545	0.04456
5	1142	4.3	93.15	7.686	0.5443	0.04433
6	2073	7.8	91.18	7.678	0.5336	0.04396
7	3432	12.9	90.00	7.710	0.5255	0.04428
8	4470	16.7	88.05	7.690	0.5135	0.04447
9	4847	18.2	86.33	7.743	0.5037	0.04475
10	4422	16.6	84.79	7.613	0.4944	0.04445
11	2924	11.0	84.05	7.736	0.4897	0.04426
12	1788	6.7	82.92	6.913	0.4837	0.03980
13	673	2.5	81.67	6.883	0.4747	0.03930
14	132	0.5	81.56	5.780	0.4773	0.03463

**Table 4:** Cardiovascular health status and the WC and WHtR.

Cardiovascular health status	n	%	WC		WHtR	
			Mean	S.D.	Mean	S.D.
Inadequate	798	3.0	95.65	7.735	0.5599	0.04443
Average	15964	59.8	88.72	7.988	0.5179	0.04621
Optimum	9939	37.2	83.98	7.521	0.4895	0.04351

**Table 5:** Correlation coefficient between cardiovascular health and WC, WHtR.

	The cardiovascular health score	The number of ideal cardiovascular health metrics	Cardiovascular health status
WC	−0.387*	−0.384**	−0.319**
WHtR	−0.400*	−0.395**	−0.330**

^*^Pearson correlation coefficient; ^**^Spearman correlation coefficient.

## References

[b1] Writing Group Members *et al*. Heart disease and stroke statistics–2010 update: a report from the American Heart Association. Circulation 121, e46–e215 (2010). 20019324 10.1161/CIRCULATIONAHA.109.192667

[b2] National Center for Cardiovascular Disease, China. Report on Cardiovascular disease in China (2013) Beijing, Encyclopedia of China Publishing House (2014).

[b3] Zhu Z. Obesity-related cardiovascular risk and its appropriate intervention. Chin J Endocrinol Metab 27, 707–710 (2011).

[b4] Matsuzawa Y. Establishment of a concept of visceral fat syndrome and discovery of adiponectin. Proc Jpn Acad Ser B Phys Biol Sci 86, 131–141 (2010). 10.2183/pjab.86.131 PMC3417563 20154470

[b5] Zhu S. . Waist circumference and obesity-associated risk factors among whites in the third National Health and Nutrition Examination Survey: clinical action thresholds. Am J Clin Nutr 76, 743–9 (2002). 12324286 10.1093/ajcn/76.4.743

[b6] Hsieh S. D., Yoshinaga H. & Muto T. Waist-to-height ratio, a simple and practical index for assessing central fat distribution and metabolic risk in Japanese men and women. Int J Obes Relat Metab Disord 27, 610–6 (2003). 12704405 10.1038/sj.ijo.0802259

[b7] Kulshreshtha A. . Life’s Simple 7 and risk of incident stroke: the reasons for geographic and racial differences in stroke study. Stroke 44, 1909–14 (2013). 23743971 10.1161/STROKEAHA.111.000352 PMC3816734

[b8] Bi Y. . Status of cardiovascular health in Chinese adults. J Am Coll Cardiol 65, 1013–25 (2015). 25766949 10.1016/j.jacc.2014.12.044

[b9] Ford E. S., Greenlund K. J. & Hong Y. Ideal cardiovascular health and mortality from all causes and diseases of the circulatory system among adults in the United States. Circulation 125, 987–995 (2012). 22291126 10.1161/CIRCULATIONAHA.111.049122 PMC4556343

[b10] Zeng Q. . Ideal cardiovascular health in Chinese urban population. Int J Cardiol 167, 2311–7 (2013). 22727977 10.1016/j.ijcard.2012.06.022

[b11] Shay C. M. . Status of cardiovascular health in US adults: prevalence estimates from the National Health and Nutrition Examination Surveys (NHANES) 2003-2008. Circulation 125, 45–56 (2012). 22095826 10.1161/CIRCULATIONAHA.111.035733 PMC4077723

[b12] Lu Y. . Prevalence of ideal cardiovascular health in southeast Chinese adults. Int J Cardio 184, 385–387 (2015). 10.1016/j.ijcard.2015.02.104 25745989

[b13] Lloyd-Jones D. M. . Defining and setting national goals for cardiovascular health promotion and disease reduction: the American Heart Association’s strategic Impact Goal through 2020 and beyond. Circulation 121, 586–613 (2010). 20089546 10.1161/CIRCULATIONAHA.109.192703

[b14] Dong C. . Ideal cardiovascular health predicts lower risks of myocardial infarction, stroke, and vascular death across whites, blacks, and hispanics: the northern Manhattan study. Circulation 125, 2975–84 (2012). 22619283 10.1161/CIRCULATIONAHA.111.081083 PMC3396556

[b15] Wu S. . Prevalence of ideal cardiovascular health and its relationship with the 4-year cardiovascular events in a northern Chinese industrial city. Circ Cardiovasc Qual Outcomes 5, 487–93 (2012). 22787064 10.1161/CIRCOUTCOMES.111.963694

[b16] Folsom A. R. . Community prevalence of ideal cardiovascular health, by the American Heart Association definition, and relationship with cardiovascular disease incidence. J Am Coll Cardiol 57, 1690–6 (2011). 21492767 10.1016/j.jacc.2010.11.041 PMC3093047

[b17] Chen C. M. . The role of dietary factors in chronic disease control in China. Obes Rev 9, Suppl 1, 100–3 (2008). 10.1111/j.1467-789X.2007.00448.x 18307709

[b18] Bi Z. . Hypertension Prevalence, Awareness, Treatment, and Control and Sodium Intake in Shandong Province, China: Baseline Results From Shandong–Ministry of Health Action on Salt Reduction and Hypertension (SMASH), 2011. Prev Chronic Dis 11, 130423 (2014). 10.5888/pcd11.130423 PMC4032056 24854239

[b19] Carr M. C. & Brunzell J. D. Abdominal obesity and dyslipidemia in the metabolic syndrome: importance of type 2 diabetes and familial combined hyperlipidemia in coronary artery disease risk. J Clin Endocrinol Metab 89, 2601–7 (2004). 15181030 10.1210/jc.2004-0432

[b20] Chinese Society of Endocrinology. The expert consensus on Chinese adult obesity prevention and control. Chin J Endocrinol Metab 27, 711–717 (2011).

[b21] Gelber R. P. . Measures of obesity and cardiovascular risk among men and women. J Am Coll Cardiol 52, 605–615 (2008). 18702962 10.1016/j.jacc.2008.03.066 PMC2671389

[b22] Lu M., Ye W., Adami H. O. & Weiderpass E. Prospective study of body size and risk for stroke amongst women below age 60. J Intern Med 260, 442–450 (2006). 17040250 10.1111/j.1365-2796.2006.01706.x

[b23] Han T. S., van, Leer E. M., Seidell J. C. & Lean M. E. Waist circumference action levels in the identification of cardiovascular risk factors: prevalence study in a random sample. BMJ 311, 1401–1405 (1995). 8520275 10.1136/bmj.311.7017.1401 PMC2544423

[b24] Lee J. S., Aoki K., Kawakubo K. & Gunji A. A study on indices of body fat distribution for screening for obesity. J Occup Health 37, 9–18 (1995). 10.1539/sangyoeisei.37.9 7780864

[b25] Hsieh S. D. & Yoshinaga H. Waist/height ratio as a simple and useful predictor of coronary heart disease risk factors in women. Inter Med 34, 1147–1152 (1995). 10.2169/internalmedicine.34.1147 8929639

[b26] Hsieh S. D. & Yoshinaga H. Abdominal fat distribution and coronary heart disease risk factors in men-waist/height ratio as a simple and useful predictor. Int J Obes Relat Metab Disord 9, 585–9 (1995). 7489031

[b27] Taylor R. W., Keil D., Gold E. J. & Goulding A. Body mass index, waist circumference girth, and waist-to-hip circumference ratio as indexes of total and regional adiposity in woman: evaluation using receiver operating characteristic curve. Am J Clin Nutr 67, 44–49 (1998). 9440374 10.1093/ajcn/67.1.44

[b28] Murphy N. F. . Long-term cardiovascular consequences of obesity: 20-years follow-up of more than 15000 middle-aged men and women (the Renfrew-Paisley study). Eur Heart J 27, 96–106 (2006). 16183687 10.1093/eurheartj/ehi506

[b29] Ashwell M., Gunn P. & Gibson S. Waist-to-height ratio is a better screening tool than waist circumference and BMI for adult cardiometabolic risk factors: systematic review and meta-analysis. Obes Rev 13, 275–86 (2012). 22106927 10.1111/j.1467-789X.2011.00952.x

[b30] Browning L. M., Hsieh S. D. & Ashwell M. A systematic review of waist-to-height ratio as a screening tool for the prediction of cardiovascular disease and diabetes: 0·5 could be a suitable global boundary value. Nutr Res Rev 23, 247–69 (2010). 20819243 10.1017/S0954422410000144

[b31] Mannucci E. . Indexes of abdominal adiposity in patients with type 2 diabetes. J Endocrinol Invest 27, 535–40 (2004). 15717650 10.1007/BF03347475

[b32] He Y., Zeng Q., Tian J., Chen Z. & Zhao X. Waist-to-height ratio as a predictor of dyslipidemia for Chinese adults. Chin J Health Manage 7, 9–13 (2013).

[b33] Tseng C. H. . Optimal anthropometric factor cutoffs for hyperglycemia, hypertension and dyslipidemia for the Taiwanese population. Atherosclerosis 210, 585–9 (2010). 20053403 10.1016/j.atherosclerosis.2009.12.015

[b34] Lin W. Y. . Optimal cut-off values for obesity: using simple anthropometric indices to predict cardiovascular risk factors in Taiwan. Int J Obes Relat Metab Disord 26, 1232–8 (2002). 12187401 10.1038/sj.ijo.0802040

[b35] Ho S. Y., Lam T. H. & Janus E. D. & Hong Kong Cardiovascular Risk Factor Prevalence Study Steering Committee. Waist to stature ratio is more strongly associated with cardiovascular risk factors than other simple anthropometric indices. Ann Epidemiol 13, 683–91 (2003). 14599732 10.1016/s1047-2797(03)00067-x

[b36] Kim S. H., Choi H., Won C. W. & Kim B. S. Optimal Cutoff Points of Anthropometric Parameters to Identify High Coronary Heart Disease Risk in Korean Adults. J Korean Med Sci 31, 61–6 (2016). 26770039 10.3346/jkms.2016.31.1.61 PMC4712581

[b37] Guasch-Ferré M. . Waist-to-height ratio and cardiovascular risk factors in elderly individuals at high cardiovascular risk. PLoS One 7, e43275 (2012). 22905246 10.1371/journal.pone.0043275 PMC3419167

[b38] Ashwell M. & Hsieh S. D. Six reasons why the waist-to-height ratio is a rapid and effective global indicator for health risks of obesity and how its use could simplify the international public health message on obesity. Int J Food Sci Nutr 56, 303–7 (2005). 16236591 10.1080/09637480500195066

[b39] Grundy S. M. . Diagnosis and management of the metabolic syndrome: an American Heart Association/National Heart, Lung, and Blood Institute scientific statement. Curr Opin Cardiol 21, 1–6 (2006). 16355022 10.1097/01.hco.0000200416.65370.a0

[b40] Zimmet P., Magliano D., Matsuzawa Y., Alberti G. & Shaw J. The metabolic syndrome: a global public health problem and a new definition. J Atheroscler Thromb 12, 295–300 (2005). 16394610 10.5551/jat.12.295

[b41] Cooperative Meta-analysis Group of China Obesity Task Force. Predictive values of body mass index and waist circumference to risk factors of related diseases in Chinese adult population. Chin J Epidemiol 23, 5–10 (2002). 12015100

[b42] Examination Committee of Criteria for ‘Obesity Disease’ in Japan & Japan Society for the Study of Obesity. New criteria for ‘obesity disease’ in Japan. Circ J 66, 987–992 (2002). 12419927 10.1253/circj.66.987

